# Panhypopituitarism in a Patient with Burkitt Lymphoma: A Diagnostic and Therapeutic Challenge

**DOI:** 10.17925/EE.2024.20.1.11

**Published:** 2023-12-15

**Authors:** Augusto Dextre-Espinoza, Sofía Pilar Ildefonso-Najarro, Marcio José Concepción-Zavaleta, Juan Eduardo Quiroz-Aldave, Diana Carolina Deutz-Gómez Condori, Fiorella Beatriz Gonzales-Chiroque, Rodrigo Martín Rodríguez-Solis

**Affiliations:** 1. Division of Endocrinology, Hospital Nacional Guillermo Almenara Irigoyen, Lima, Perú; 2. Universidad Científica del Sur, Lima, Perú; 3. Division of Non-communicable Diseases, Endocrinology Research Line, Hospital de Apoyo Chepén, Chepén, Perú

**Keywords:** Burkitt lymphoma, HIV infection, hypopituitarism, lymphatic metastasis, neuroendocrinology, pituitary gland, pituitary infiltration

## Abstract

Pituitary infiltration by systemic lymphoma is an exceedingly rare occurrence. Given its high mortality rate, it is crucial to recognize its clinical, biochemical and radiological features in order to provide timely intervention. We present the case of a 26-year-old male with a history of human immunodeficiency virus (HIV) infection who presented to the hospital with severe anemia, persistent fever, weight loss and diarrhea over the previous 4 months. Physical examination revealed a compromised general condition, fever, pallor, hepatomegaly and lymphadenopathy. Cervical lymph node biopsy confirmed Burkitt lymphoma (BL). During hospitalization, the patient developed polyuria, polydipsia, hypernatremia, fluid-resistant hypotension and hypoglycaemia. Corticosteroid therapy was initiated due to suspected adrenal insufficiency, resulting in clinical improvement but exacerbation of polyuria and hypernatremia. Plasma and urinary osmolarity confirmed arginine vasopressin deficiency, and assessment of anterior pituitary reserve revealed hypopituitarism, necessitating hormonal replacement therapy. Sellar magnetic resonance imaging with contrast revealed pituitary infiltration. The patient subsequently developed septic shock and died. BL accounts for approximately 10% of the cases of pituitary infiltration associated with lymphoma. Clinical presentation is heterogeneous, with panhypopituitarism often serving as the initial manifestation. Sellar magnetic resonance imaging plays a pivotal role in the differential diagnosis. Management typically entails chemotherapy, immunotherapy, radiation and hormonal replacement therapy. This case report describes a patient with BL and HIV infection who developed panhypopituitarism due to pituitary infiltration, an exceedingly rare presentation considered a medical emergency.

Burkitt lymphoma (BL) is a non-Hodgkin B-cell lymphoma originating from the germinal center, characterized by dysregulation of the *MYC* gene, often resulting from the translocation of chromosome 8 into 14. It is extremely aggressive, representing the fastest proliferating cancer, and typically involves the lymph nodes, bone marrow and the central nervous system.^1,2^ Three identifiable subtypes include sporadic, endemic, and immunodeficiency-associated BL. The latter manifests in patients with human immunodeficiency virus (HIV), congenital immunodeficiencies and recipients of allograft transplants.^1^

Pituitary metastases (PM) are exceedingly rare, and it is even more unusual for them to be secondary to haematologic malignancies, with systemic lymphoma being the primary cause in these instances, accounting for 0.5% of all PM cases.^3,4^ From a histological perspective, non-Hodgkin B-cell lymphomas are the most common lymphomas to cause PM. To date, the medical literature has documented 34 cases of pituitary infiltration by lymphomas.^3^

The clinical presentation is heterogeneous, but there is usually rapid tumor growth and dissemination to other organs, resulting in a poor prognosis for affected cases.^5^ PM can affect both the adenohypophysis, which can primarily trigger hypothyroidism and central adrenal insufficiency, as well as the neurohypophysis, which can lead to arginine vasopressin (AVP) deficiency.^3^ Panhypopituitarism is reported to be even rarer.^6^

The purpose of this case report is to present the case of a male patient with HIV infection and BL who developed panhypopituitarism due to PM. This form of presentation is extremely rare, and it is essential to recognize its clinical and biochemical characteristics to initiate prompt treatment, given its high risk of mortality.

## Case report

A 26-year-old male Peruvian patient initially presented with episodes of diarrhea without mucus and blood associated with hyporexia, a 5 kg weight loss, intermittent fever and asthenia over a 4-month period. Two months later, he developed a vesicular mucocutaneous rash on his lips and a productive cough. These symptoms led to a diagnosis of HIV infection, with a CD4 T lymphocyte count of 119 cells/mm^3^, and he was started on antiretroviral treatment.

One month after initiating treatment, his symptoms worsened significantly. He experienced dyspnea upon moderate exertion, palpitations and a holocranial headache, prompting him to seek care in an emergency department. On physical examination, he displayed tachycardia (heart rate: 150 beats per minute), an axillary temperature of 38 °C, and marked weight loss (body mass index: 18.2 kg/m^2^). He exhibited pallor and had palpable 1x1.5 cm hard lymph nodes in the right cervical region, as well as bilateral inguinal lymphadenopathy and hepatomegaly. Severe anemia (haemoglobin: 6.5 g/dL) was observed upon admission, necessitating transfusion therapy.

A contrast-enhanced thoracoabdominal computed tomography (CT) scan revealed bilateral laminar pleural effusion, multiple mediastinal lymph nodes and a conglomerate of right cervical lymph nodes, in addition to hepatomegaly and splenomegaly. Broad-spectrum antibiotic therapy was initiated due to a positive bone marrow culture for coagulase-negative *Staphylococcus*, with intravenous (IV) linezolid 600 mg every 12 hours (h) and IV meropenem 2 g every 8 h.

Bone marrow aspiration indicated infiltration of immature cells and multiple cytoplasmic and intranuclear vacuoles, raising suspicion of BL or acute lymphocytic leukemia. Flow cytometry and cervical lymph node biopsy confirmed the diagnosis of BL.

During hospitalization, the patient experienced polyuria (5 L/day), polydipsia, and hypernatremia (sodium (Na): 148 mmol/L). Subsequently, the patient developed hypotension (blood pressure: 89/53 mmHg) unresponsive to fluid therapy, hypoglycaemia (glucose: 69 mg/dL), and metabolic acidosis with an increased anion gap. Due to suspected adrenal insufficiency, treatment with IV hydrocortisone 100 mg every 8 h was initiated, resulting in clinical improvement. However, polyuria worsened to 12 L/day, accompanied by hypernatremia (Na: 154 mmol/L) after initiating corticosteroid therapy. Serum osmolality was 302 mOsm/ kg, and urine osmolality was 70 mOsm/kg, confirming the diagnosis of AVP deficiency.

A contrast-enhanced sellar magnetic resonance imaging (MRI) revealed a heterogeneous appearance of the pituitary with a 6 mm thickening of the pituitary stalk, loss of brightness in the neurohypophysis, and isointensity on T2-weighted images. No signs of parasellar or suprasellar involvement were observed, consistent with pituitary infiltration.

Due to the patient's septic condition, IV corticosteroid therapy with hydrocortisone 100 mg every 8 h was continued. Additionally, intranasal desmopressin was initiated at a dose of 2 puffs every 24 h, leading to an improvement in polyuria.

Evaluation of anterior pituitary function revealed involvement of the thyroid and gonadal axes (*Table 1*), and as part of hormone replacement therapy, levothyroxine 100 µg orally every 24 h and intramuscular testosterone enanthate 250 mg every 15 days were added.

**Table 1:** Laboratory results during patient hospitalization

Laboratory test (unit of measurement)	Result	Reference range*
***Haematology***		
Leucocytes (x10^3^/µL)	8.46	4.00–11.00
Haemoglobin (g/dL)	6.5	11.5–16.5
Platelets (x10^3^/µL)	323.000	150.000–400.000
***Biochemistry***		
Urea (mg/dL)	22	19–43
Sodium (mmol/L)	148	135–150
Potassium (mmol/L)	2.56	3.50–5.00
Creatinine (mg/dL)	0.56	0.60-1.20
Total protein (g/dL)	3.5	6.3–8.2
Albumin (g/dL)	1.7	3.5–5.0
Bilirubin (mg/dL)	0.12	≤2.0
ALP (UI/L)	280	38–126
ALT (UI/L)	78	≤33
AST (UI/L)	25	≤31
LDH (UI/L)	1,535	140–271
Serum osmolality (mOsm/kg)	302	275–295
Urine osmolality (mOsm/ kg)	70	50–1,200
***Hormonal profiles***		
Total testosterone (ng/dL)	29.1	123–813
Free thyroxine (pmol/L)	0.54	11.80–23.20
TSH (mU/L)	2.53	0.40–5.50
IGF-1 (µg/L)	35.4	130–295

*Reference ranges provided by the hospital laboratory

*ALP = alkaline phosphatase; ALT = alanine aminotransferase; AST = aspartate aminotransferase; IGF-1 = insulin-like growth factor 1; LDH = lactate dehydrogenase; TSH = thyroid-stimulating hormone.*

The patient's clinical condition was attributed to pituitary infiltration by BL, as he presented with a systemic clinical syndrome of lymphoma along with panhypopituitarism, obviating the need for a pituitary biopsy. Unfortunately, the patient passed away 2 weeks after diagnosis due to septic shock, without the opportunity to receive chemotherapy.

## Discussion

PM typically occur in patients with an average age of around 60 years, with no significant differences between men and women.^7^ Pituitary infiltration by systemic lymphoma is an exceedingly rare phenomenon, accounting for only 0.5% of cases.^3^ This is in contrast to cases secondary to solid tumors, which account for 60% of PM.^4^ The most frequent primary tumor sites for these cases include the lung (28%), breast (19%), kidney (15%), thyroid (8%), gastrointestinal tract (6%) and prostate (6%).^7^

The age of cases of PM secondary to lymphoma ranged from 39 to 78 years in women and from 19 to 77 years in men, with a male predominance (2:1).^3^ In our case, the patient's age was 26 years, highlighting the unusual presentation of this disease. The average time elapsed between the diagnosis of the primary cancer and the detection of PM is 6.5 months, although in nearly half of the cases, they are diagnosed simultaneously.^7^

**Table 2:** Reported cases of secondary pituitary metastases due to Burkitt lymphoma

Age	Sex	Pituitary function	PRL	Symptoms	Imaging	Primary neoplasm	Treatment	Other	Outcome	Reference (year)
15 years	Male	Panhypopituitarism	Elevated	Stationarily in prepubertal state	9 mm enhancing lesion in the pituitary stalk	CNS Burkitt lymphoma	Chemotherapy	AVP deficiency, family history of malignancy	Remission	Silfen (2001)^11^
57 years	Male	Panhypopituitarism	Elevated	Fever and abdominal pain	Thickened pituitary stalk	Abdominal Burkitt lymphoma	Chemotherapy	Diffuse large B-cell lymphoma	Deceased	Tan (2013)^12^
26 years	Male	Panhypopituitarism	NS	Polyuria, polydipsia	Small pituitary fossa	Renal Burkitt lymphoma	Chemotherapy	NS	Remission	Yang (2013)^5^
39 years	Female	Panhypopituitarism	Elevated	Cranial nerve palsies, headache, vomiting, polyuria	Widened sella turcica with an enhancing mass lesion of 1.6 x 1.4 cm	Jaw Burkitt lymphoma	Chemotherapy	NS	Deceased	FOO (2014)^6^
8 years	Male	Panhypopituitarism	Elevated	Headache, vomiting, polyuria	Mass (52 x 54x61 mm) in the posterior wall of the nasal cavity, infiltrating the sella turcica	Nasopharyngeal Burkitt lymphoma	Chemotherapy	NS	Remission	Garda (2022)^13^

*AVP = arginine vasopressin; CNS = centrai nervous system; NS = not specified; PRL = proiactin.*

Approximately 90% of PM cases secondary to lymphoma are attributed to non-Hodgkin lymphoma, with the most common form being diffuse large B-cell lymphoma, accounting for approximately 50% of cases, followed by BL, which constitutes around 10%.^4,5,8^ In our case, the aetiology of PM was BL.

BL may present with elevated serum lactate dehydrogenase levels, indicating tumor lysis.^2^ Headache, constitutional symptoms, or syndromes related to cranial nerves or the optic chiasm may occasionally manifest later, but progress rapidly.^5,9,10^ However, in the presented case, the patient did not exhibit symptoms of compression.

The symptoms of PM tend to be non-focal and nonspecific, such as fatigue and headache. Additionally, they may present symptoms similar to those of primary pituitary lesions, including visual dysfunction and endocrine abnormalities in 65% of patients, half of whom experience panhypopituitarism.^7^ Most patients exhibit pituitary dysfunction before systemic lymphoma becomes evident, however, in other cases, lymphoma can precede pituitary dysfunction or develop during the course of the disease, as was the case with our patient, whose pituitary symptoms were documented during hospitalization.^3,5,6^

Previous reports suggested that PM had a predilection for the posterior gland, possibly due to its high vascularity, which would facilitate the distribution of cancer cells, as well as the smaller size of the posterior pituitary, allowing smaller volume metastases to affect a higher percentage of normal tissue, leading to hormonal dysfunction.^4,5,9^ However, a scoping review found that cases of panhypopituitarism were 34% more frequent than cases of AVP deficiency, and even identified cases of hypopituitarism without AVP deficiency. There may be underdiagnosis of anterior pituitary dysfunction, as its symptoms could be incorrectly attributed to systemic lymphoma.^7^

The most common endocrine disorders associated with lymphomatous PM include central hypothyroidism (67%), central adrenal insufficiency (61%), AVP deficiency (58%), hypogonadism (54%) and hyperprolactinemia (29%).^3^ All reported cases of PM secondary to BL exhibited panhypopituitarism, and the majority also had hyperprolactinemia, as illustrated in *Table 2*.^5,6,11–13^ In the present case, the patient presented with panhypopituitarism at the time of diagnosis.

The staging of BL requires biopsy and bone marrow aspiration, cerebrospinal fluid analysis, whole-body CT, and, in cases of extranodal disease, a positron emission tomography scan. Additionally, in patients with neurological symptoms, MRI of the brain and/or spinal cord is recommended.^2^ In the case of our patient, pituitary infiltration is considered stage IV.^14^

**Figure 1:**
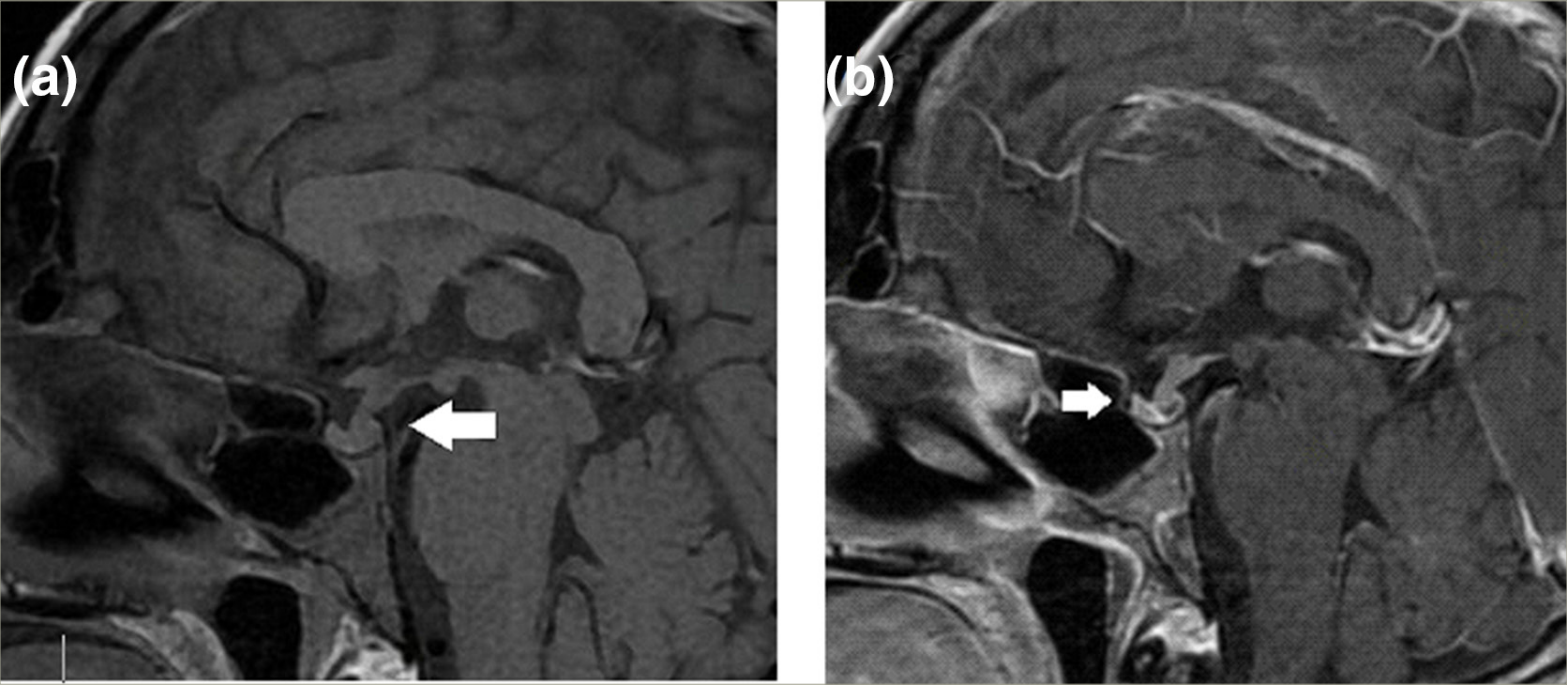
Pituitary magnetic resonance imaging

Differential imaging diagnosis is crucial in these cases. While there are no specific findings for pituitary infiltration by lymphoma on MRI, the most commonly reported characteristic is heterogeneous enhancement of the posterior lobe and the pituitary stalk following contrast administration, compared with the more homogeneous enhancement pattern observed in pituitary adenomas and primary pituitary lymphomas.^7,15^ Additionally, the absence of the normal posterior pituitary signal on T1-weighted images may indicate involvement of the posterior lobe, aiding in the differentiation between metastases and adenomas. Tumor growth rate and destruction of the sella turcica are indicative of malignancy. Oedema along the optic chiasm and optic tract is another sign found in PM, which is uncommon in pituitary adenomas.^7^ In the reported case, the MRI findings were suggestive of pituitary infiltration (*Figure 1*).

In patients with systemic lymphoma and MRI suggestive of PM and presenting with symptoms of hypopituitarism, it is rarely necessary to perform a pituitary gland biopsy to confirm lymphoma infiltration. Diagnosis is mostly based on the correlation of clinical and imaging characteristics, as in the case we have presented, and therapeutic response can serve as confirmation, since the volume of PM will rapidly decrease after systemic chemotherapy, which does not occur in pituitary adenomas, apoplexy or hypophysitis.^3,16^

BL treatment is primarily based on chemotherapy, along with supportive measures.^2^ PM management is multidisciplinary and involves surgery, radiation therapy (partial or whole-brain radiotherapy, or stereotactic radiosurgery), chemotherapy, and immunotherapy, along with the replacement of necessary hormones.^7^

Despite the available options, the treatment of PM is generally considered palliative, with an average survival ranging from 6 to 12.9 months. No treatment has been identified to significantly improve patient prognosis. Factors such as primary histology, control of systemic disease, the presence of multiple metastases, a short interval between the diagnoses of primary cancer and PM, and advanced age all influence prognosis.^7^

*a). T1 without contrast in sagittal section, intrasellar pituitary gland with absence of T1 hyperintensity in the neurohypophysis (arrow) and thickening of the infundibulum (6 mm). (b). T1 in sagittal section with heterogeneous enhancement after contrast administration.*

The prognosis for patients with PM by lymphoma is unfavorable; mortality has been reported in 44% of patients, and it is often attributed to sepsis, as in the reported case.^3,10^ Complete remission of pituitary dysfunction has been reported in 12% of cases, although long-term follow-up in these patients is typically limited due to the high associated mortality and the fact that complete restoration of pituitary function may take more than 2 years.^3,17^ This could not be assessed in our case, as the patient passed away before receiving treatment for BL.

## Conclusions

BL is a medical emergency and requires prompt treatment. PM from BL are extremely rare and may be diagnosed late, especially if they are not associated with obvious signs and symptoms of systemic lymphoma. It is essential to maintain a high index of suspicion in patients presenting with pituitary dysfunction, rapidly evolving neurological manifestations, and characteristic radiological findings.

## Limitations

The reported case has limitations, including the inability to measure prolactin and the fact that the baseline cortisol was measured with corticosteroid treatment. This was due to the patient's clinical condition, which made it impossible to request a baseline cortisol measurement.
